# Social Network and Environment as Determinants of Disability and Quality of Life in Aging: Results From an Italian Study

**DOI:** 10.3389/fmed.2022.854779

**Published:** 2022-05-23

**Authors:** Erika Guastafierro, Claudia Toppo, Barbara Corso, Rosa Romano, Rino Campioni, Ersilia Brambilla, Carla Facchini, Sara Bordoni, Matilde Leonardi

**Affiliations:** ^1^Neurology, Public Health, Disability Unit, Fondazione IRCCS Istituto Neurologico Carlo Besta, Milan, Italy; ^2^CNR, Neuroscience Institute, Padova, Italy; ^3^Auser Regionale Lomabardia, Milan, Italy; ^4^Department of Sociology, University of Milano-Bicocca, Milan, Italy; ^5^Associazione Nestore, Milan, Italy

**Keywords:** aging, social network, environment, health, functioning, disability, quality of life

## Abstract

**Background:**

The increase in life expectancy is leading to a worldwide increase in chronic diseases and disability, with significant concern about their management and long-term care. Investigating the aging process using a bio-psychosocial perspective is essential to understanding how to reduce disability and improve the quality of life of aging people. This study aims to explore the role of social networks and built environment as predictors of disability and quality of life in the Italian population aged over 50 years.

**Materials and Methods:**

The research protocol is composed of several tools: World Health Organization Disability Assessment Scale 2.0 (WHODAS 2.0), World Health Organization Quality of Life Assessment in Aging (WHOQOL-AGE), Social Network Index (SNI), the Courage Built Environment Self-Reported Questionnaire (CBE-SR), and collection of sociodemographic information and information on health system coverage.

**Results:**

A total of 431 people were administered the protocol, and among them, 209 were males and 222 were females, with a mean age of 70 years. The majority of the sample reported earning a middle or high school diploma, and 60.6% of the sample declared to have a good health status. The results showed that people with a good social support network have higher levels of functioning and quality of life. However, the built environment did not significantly predict either disability or quality of life.

**Conclusions:**

These results could provide elements for dialogue with institutions and policymakers. This is fundamental to develop active policies aimed at the implementation of services and systems to promote healthy aging process.

## Introduction

The aging population of the world will grow rapidly in the coming decades (1). In Europe, the percentage of people aged 65+ years was around 21% in 2020 [https://ec.europa.eu/eurostat/cache/digpub/demography/ (Accessed December 17, 2021)]. Italy is one of the European countries where the percentage of people aged 65+ years is among the highest, which was recorded to be 23.4% in 2021 [https://www.istat.it/it/popolazione-e-famiglie?dati (Accessed December 17, 2021)].

The aging population, with increasing life expectancy and morbidity and thus increasing disability levels, poses major challenges for the traditional social welfare states, due to the greater need for health and social care for older people (2).

In the past years, a growing body of epidemiological and research studies tried to evaluate determinants of disability in aging populations to find out what needs to be done to promote healthy aging. The World Health Organization (WHO) defines the term healthy aging as the process to develop and maintain the functional ability which can enable well-being in older age (3) with preservation of good physical and cognitive function, along with high level of independence and active engagement within the wider society (4).

The WHO uses the term “functional ability” to indicate the result of the interaction between the individual intrinsic capacity, which comprises all the physical and mental abilities, such as cognition and mobility, and the environment that can act as a barrier or a facilitator (3). The functional ability includes several domains: the ability to meet basic needs, the ability to learn, grow, and make decisions, mobility, the ability to build and maintain relationships, and the ability to contribute to society (5).

According to the bio-psychosocial model (6), which considers the influence of biological, psychological, and social factors in determining health and disease, functioning, and disability are the results of the person–environment interaction. The environmental factors include both the social and the physical environment (7). Therefore, it is important to investigate the role of these factors, particularly for older adults who are more likely to experience limitations in their intrinsic capacity due to the presence of one or more health conditions.

Another key concept to observe healthy aging is the quality of life (QoL), which is defined by WHO as “an individual's perception of their position in life in the context of the culture and value systems in which they live and in relation to their goals, expectations, standards, and concerns.” [https://www.who.int/tools/whoqol (Accessed March 14, 2022)].

Different research studies have already outlined the role of social networks and the built environment as the determinants of healthy aging (8–10). Specifically, some authors have focused on the analysis of the built environment to assess the presence of facilitators or barriers that can influence the healthy aging process (11). For example, some studies, based on the idea that it is important for older people to stay at home as long as possible (the concept of “aging in place”), believe that it is necessary to adapt the home environment to the needs emerging with aging, taking into account safety and security and also the livability and the pleasantness of the environment (12–15). A study conducted in Sweden (16) showed the presence of environmental barriers both in multi-dwellings and in one-family houses. This study has highlighted, through a functional profile analysis, that the environmental barriers found in older houses will cause accessibility difficulties also in aging people with few functional limitations. More evidence (16–20) showed that accessibility in houses depends on activities of daily living (ADL), falls, and institutionalization, restricting social participation which further results in higher demand for health care support (21). Moreover, a safe, accessible, and age-friendly built environment in the neighborhood and, more generally, in cities and towns can prevent injuries and support mobility independence. In addition, it promotes active participation and engagement in community life (22, 23), particularly considering that older adults spend more time in the neighborhood compared to the younger adults; therefore, neighbors can become important social contacts and facilitate inclusion (24).

Indeed, as documented in several studies (25–27), social participation and integration in social life and activities have a positive impact on functioning and QoL in older adults. On the contrary, loneliness is related to an increase in negative emotions and a decrease in life satisfaction, which can further lead to depression and thus bad health (9, 28). Unfortunately, loneliness is commonly experienced by elderly people. With the retirement from work, people find themselves in a new daily life emptied of the activities they have carried out throughout their lives and out of a social network with shared interests and activities. Moreover, with the progress of age, it becomes more likely for older adults to lose their loved ones, such as their partner, family members, and close friends (9, 23, 28). In a study conducted on the types of social networks (29), aging people living alone had a higher probability to create a restricted social network (e.g., lower number of kin and non-kin membership, low intensity of contacts, and few geographically close social network members). This study also showed that it is more probable to find this type of closer network in Eastern and Southern European countries, such as Slovenia, Italy, and Poland.

Considering the influence of the environment and social networks on disability and QoL in aging people, it is necessary to explore to what extent these factors can contribute to the increase and maintenance of a high functioning level (28). The present study aims to explore the role of social networks and built environment as the predictors of disability and QoL in the Italian population, aged over 50 years, residing in the Lombardy Region.

## Materials and Methods

### Sample and Procedure

Within a grant supported by Cariplo Foundation, a sample of 431 participants aged 50+ years were enrolled and agreed to participate in the study. Inclusion criteria for participation in the study were speaking Italian, being a resident of the Lombardy Region (the most populated region of Italy), and volunteering in different roles in the national association Auser, which is devoted to older people in Italy and has 45,000 volunteers. Persons were recruited from Auser centers located in Lombardy Region, thanks also to the collaboration in the recruitment of the Auser referent in each site. Persons were interviewed face to face by 14 interviewers trained on the administration and use of the CAPI protocol and in the management of the data collection platform on tablet support.

Data were collected from January 2020 to May 2021, that is, during the peak of the COVID-19 emergency. Face-to-face interviews were conducted with respect to the safety recommendations put forward by the Italian Ministry of Health, such as maintaining a social distance of 2 m, disinfection of space and materials, and use of a face mask and a plexiglass barrier between the interviewer and the interviewed. The study was designed according to the ethical standards of the Declaration of Helsinki and was approved by the Ethical Committee of the Fondazione IRCCS Istituto Neurologico Carlo Besta of Milan. Informed consent was obtained from all the participants before participation in the study.

### Measures

A set of validated tools was administered to the sample by trained interviewers. The research protocol was previously developed and used in two projects: COURAGE in Europe (30) and the Italian IDAGIT study (31). The research protocol, which proved to be a valid tool for collecting comparable data on the aging population (30), is composed of a series of tools for investigating several domains.

Chronic conditions were assessed by the self-report question, “Has a health care professional ever told you, you have...?” for the following eight conditions: arthritis, stroke, angina, diabetes, lung disease, asthma, depression, and hypertension (32). Based on the presence/absence of all these conditions, a categorical variable for non-communicable diseases (NCDs) was created and categorized as none, one, two, three, or more.

Functioning and disability were evaluated using the 12-item validated version of the World Health Organization Disability Assessment Schedule 2.0 (WHODAS 2.0) (33). The items used to obtain the WHODAS 2.0 score cover six domains (cognition, mobility, self-care, getting along, life activities, and participation) and inspect the level of difficulty that the participant had in conducting these activities in the past 30 days. Possible answers were as follows: none, mild, moderate, severe, and extreme/cannot do. The WHODAS 2.0 score was implemented by the item response theory (IRT) analysis and then normalized from 0 (no disability) to 100 (maximum disability).

For appraising cognitive functioning, a one-factor score was obtained from the items regarding verbal fluency, immediate verbal recall, delayed verbal recall, digit span backward, and digit span forward (34). The global score was then transformed into a percentile scale, with higher scores indicating better cognitive functioning.

Mobility was assessed by asking the participants how much difficulty they had, over the last 30 days, in doing 15 activities (standing for long periods, such as 30 min; climbing one flight of stairs without resting; vigorous activities; sitting for long periods; stooping, kneeling, or crouching; picking up things with fingers; extending arms above shoulder level; walking 100 m; walking a long distance, such as a kilometer; carrying things; moving around inside home; getting up from lying down; standing up from sitting down; getting where you want to go, using private or public transport if needed; and getting out of your home). Possible answers ranged from “no problem” to “complete problem/cannot do the activity.” A one-factor score was calculated as detailed in Raggi et al., 2018 (35). The score was then transformed into a percentile scale, with higher scores indicating better mobility.

Quality of life was measured by the validated 13-item instrument WHOQOL-AGE (36) score. It covers different areas considered relevant for the aging population, such as satisfaction related to health, social relationships, economic aspects, and personal growth. It provides a single score that ranges from 0 to 100, with higher scores indicating higher QoL.

The built environment was inspected by the 19 items of the Courage Built Environment Self-Reported Questionnaire (CBE-SR) (37). From the 19 items, four scores were created, covering the following aspects: “Usability of the neighborhood environment,” “Hindrance of walkable environment,” “Easiness of use of public buildings, places, and facilities,” and “Usability of the living place.” Each score was transformed into a percentile scale, with a higher score indicating a neighborhood environment perceived as more usable; a walkable environment perceived as more hindering; public buildings, places, and facilities perceived as easier to use; and a living place perceived as less risky and more usable, respectively.

The impact of social networks was evaluated through four indices. The Social Networks Index (SNI), which reveals good reliability and content validity, is described in detail elsewhere (38). It is based on a multidimensional set of independent networks [involving the relations with spouse, parents, other relatives (children, grandchildren, and others), neighbors, friends, and co-workers] taking into account the size of specific networks, the ties (close relations), help (general social support), and the frequency of face-to-face contacts. The SNI ranged from 0 to 100, with a higher score indicating a better social network.

The social support was measured by using the OSLO-3 Social Support Scale (39, 40) based on the following three questions: “How many people are so close to you that you can count on them if you have serious problems?”, “How much concern do people show in what you are doing?”, and “How easy can you get help from neighbors if you should need it?”. The scores of the last two questions were reverted in the syntax to reflect that a high score indicates high support. A global score was created by adding up the three items and transforming the result into a percentile scale, with higher scores indicating higher social support.

Loneliness was assessed by employing the three-item UCLA Loneliness Scale (41): “How often do you feel that you lack companionship?”, “How often do you feel left out?”, and “How often do you feel isolated from others?”. Responses to the three items were added up and transformed into a percentile scale, with higher scores indicating a higher subjective perception of loneliness.

A global score was developed for trust as a one-factor score based on five questions that inspect if and to what extent participants can trust family members, neighbors, co-workers, and strangers. The score was transformed into a percentile scale, with higher scores indicating a higher trust perception.

To measure participation, the act of joining with others in doing something, two-factor scores were created from eight questions regarding the propensity of the respondent to participate in several activities (e.g., attending any group, club, society, union, or organizational meeting, sports clubs, sports competitions, or visits friends or had them over his/her house). From these two scores, a global factor score for the participation section was calculated and transformed into a percentile scale, with higher scores indicating higher participation.

### Statistical Analysis

Descriptive statistics are presented overall as frequencies and percentages for categorical data, while as mean and standard deviation for continuous data.

Two stepwise multivariable forward regression models were conducted (*p*-value <0.05 for variable inclusion, and *p*-value <0.15 for variable removal) to identify the possible determinants of QoL and functioning and disability. Analysis of residuals was performed to examine models' goodness of fit and adherence to regression assumptions. Multicollinearity was checked using the tolerance and the variance inflation factor (VIF); variables with tolerance <0.4 (VIF >2.5) were discarded from the analysis. Models were adjusted for age and gender.

The significant level was set at 0.05. All statistical analyses were performed using STATA, version 15.

## Results

Table 1 presents a complete overview of the sociodemographic information of the sample. The sample (*N* = 431) was equally divided between women (*N* = 222) and men (*N* = 209), with a mean age of 69.67 years (SD = 6.65). Most of the sample consisted of retired (84.7%) and married (64.7%) people. With regard to education, the major portion of the sample earned a middle school (34.3%) or high school (47.3%) diploma. Table 2 shows the descriptive statistics of the measures used in the research protocol. Most of the sample declared to suffer from 0 to 2 diseases of the eight NCDs included in the questionnaire, and 60.6% of the participants defined their health as “good.” With respect to WHODAS 2.0 score, the sample obtained good scores in general, reflecting a low disability level, and specifically for the mobility score, the sample obtained a mean score that reflected a low level of difficulties in mobility. Also, considering the WHOQOL-AGE score, most of the sample showed a perceived good quality of life (59.59). The average score of SNI was found to be 58.20, which indicated that the majority of the sample has a good social network, specifically considering the component of social support (68.79) and a low loneliness score (14.85). Finally, the descriptive results from CBE highlighted that the majority of the sample reported that their neighborhood environment (76.52) and the place in which they live (92.09) are usable and that the public buildings and places are easy to access and use (81.93).

**Table 1:** Demographic characteristics of study participants (*N* = 431).

	**Male**	**Female**	**Total**	***p*-Value**
	**(*N* = 209)**	**(*N* = 222)**	**(*N* = 431)**	
**Age (year)**				0.006
Mean (SD)	70.56 (5.55)	68.82 (7.45)	69.67 (6.65)	
Min—Max	57.0–87.0	50.0–90.0	50.0–90.0	
**Marital status**				<0.001
Never married	6 (2.9%)	25 (11.3%)	31 (7.2%)	
Married	174 (83.3%)	105 (47.3%)	279 (64.7%)	
Cohabiting	3 (1.4%)	7 (3.2%)	10 (2.3%)	
Separated/divorced	8 (3.8%)	31 (14.0%)	39 (9.0%)	
Widow	18 (8.6%)	54 (24.3%)	72 (16.7%)	
**Education**				0.374
Primary school	11 (5.3%)	22 (9.9%)	33 (7.7%)	
Secondary school	77 (36.8%)	71 (32.0%)	148 (34.3%)	
High school	99 (47.4%)	105 (47.3%)	204 (47.3%)	
College/University	20 (9.6%)	23 (10.4%)	43 (10.0%)	
Post graduate degree	2 (1.0%)	1 (0.5%)	3 (0.7%)	

*SD, standard deviation.*

*p-value: t-test significance for quantitative variables and chi-squared significance for categorical variables*.

**Table 2:** Descriptive statistics (*N* = 431).

	**Male (*N* = 209)**	**Female (*N* = 222)**	**Total (*N* = 431)**	***p*-Value**
**Self-report health**				0.402
Very good	29 (13.9%)	22 (9.9%)	51 (11.8%)	
Good	125 (59.8%)	136 (61.3%)	261 (60.6%)	
Moderate	47 (22.5%)	48 (21.6%)	95 (22.0%)	
Bad	6 (2.9%)	8 (3.6%)	14 (3.2%)	
Very bad	2 (1.0%)	7 (3.2%)	9 (2.1%)	
Refused	0 (0.0%)	1 (0.5%)	1 (0.2%)	
**NCDs (on 8 conditions)**				0.989
No NCDs	62 (29.7%)	65 (29.3%)	127 (29.5%)	
1 NCD	74 (35.4%)	76 (34.2%)	150 (34.8%)	
2 NCDs	53 (25.4%)	59 (26.6%)	112 (26.0%)	
3+ NCDs	20 (9.6%)	22 (9.9%)	42 (9.7%)	
**WHODAS score: 0** **=** **Best 100** **=** **Worst**				0.001
Mean (SD)	5.27 (8.81)	9.07 (13.66)	7.23 (11.71)	
Min–Max	0.0–51.2	0.0–100.0	0.0–100.0	
**Cognition score 0** **=** **Worst 100** **=** **Best**				0.099
Mean (SD)	59.07 (16.38)	61.69 (16.58)	60.42 (16.51)	
Min–Max	0.0–97.0	0.0–100.0	0.0–100.0	
**Mobility score 0** **=** **Best 100** **=** **Worst**				0.003
Mean (SD)	7.32 (10.80)	11.73 (17.08)	9.53 (14.46)	
Min–Max	0.0–63.6	0.0–100.0	0.0–100.0	
*N* (% missing)	190 (9.1%)	192 (13.5%)	382 (11.4%)	
**Social support score 0** **=** **Worst 100** **=** **Best**				0.950
Mean (SD)	68.85 (17.75)	68.74 (19.22)	68.79 (18.50)	
Min–Max	10.0–100.0	0.0–100.0	0.0–100.0	
**Loneliness score 0** **=** **Best 100** **=** **Worst**				0.003
Mean (SD)	11.80 (17.34)	17.72 (23.22)	14.85 (20.77)	
Min–Max	0.0–100.0	0.0–100.0	0.0–100.0	
**Trust score 0** **=** **Worst 100** **=** **Best**				0.025
Mean (SD)	37.07 (18.46)	41.18 (19.45)	39.19 (19.07)	
Min–Max	0.0–82.1	0.3–100.0	0.0–100.0	
**Participation score** ***0** **=** **Worst 100** **=** **Best***				0.019
Mean (SD)	27.35 (21.09)	22.67 (20.29)	24.94 (20.79)	
Min–Max	0.0–100.0	0.0–76.1	0.0–100.0	
**WHOQOL-AGE 0** **=** **Worst 100** **=** **Best**				0.008
Mean (SD)	61.83 (15.31)	57.42 (17.87)	59.59 (16.79)	
Min–Max	28.3–97.9	0.0–100.0	0.0–100.0	
*N* (% Missing)	201 (3.8%)	208 (6.3%)	409 (5.1%)	
**CBE: Usability of the neighborhood environment**				0.730
Mean (SD)	76.09 (24.88)	76.92 (25.15)	76.52 (24.99)	
Min–Max	0.0–100.0	6.9–99.6	0.0–100.0	
**CBE: Hindrance of walkable environment**				0.406
Mean (SD)	22.27 (22.89)	24.12 (23.28)	23.23 (23.08)	
Min-Max	0.0–100.0	0.0–100.0	0.0–100.0	
**CBE: Easiness of use of public buildings, places and facilities**				0.273
Mean (SD)	80.79 (21.14)	83.01 (20.70)	81.93 (20.92)	
Min–Max	0.0–100.0	8.5–100.0	0.0–100.0	
**CBE: Usability of the living place**				0.136
Mean (SD)	93.21 (12.50)	91.04 (17.12)	92.09 (15.08)	
Min–Max	24.3–100.0	0.0–100.0	0.0–100.0	

*SD, standard deviation; NCD, non-communicable diseases; WHODAS, World Health Organization Disability Assessment Schedule; WHOQOL-AGE, World Health Organization Quality of Life; CBE, Courage Built Environment.*

*p-value: t-test significance for quantitative variables and chi-squared significance for categorical variables*.

Table 3 shows the final regression model considering QoL score, measured through the WHOQOL-AGE instrument, as a dependent variable, adjusted for age and gender. The most significant predictors of QoL were found to be the Social Network Index, Loneliness Scale, and Disability Scale (measured using the WHODAS 2.0). In particular, higher scores in the Social Network Index predicted an increase in quality of life, while higher scores in the Loneliness and Disability scales predicted a decrease in quality of life.

**Table 3:** Final regression model considering Quality of Life score as dependent variable, measured through the WHOQOL-AGE instrument, adjusted for age and gender.

**Variable**	**Coefficient**	**standard error**
Loneliness score (0 = best – 100 = bad)	−0.185***	0.039
Social Network Index (0 = bad – 100 = best)	0.235***	0.057
WHODAS score (0 = best – 100 = bad)	−0.182***	0.070
Constant	71.720***	8.973
No. of Observation	395	
*R*^2^ (Adjusted *R*^2^)	0.18 (0.17)	

*WHOQOL-AGE, World Health Organization Quality of Life; WHODAS, World Health Organization Disability Assessment Schedule.*

*** p <0.01, ** p <0.05, * p <0.1.

It should be noted that in the present model, the value of *R*^2^ was found to be low (0.18), probably because the additional variables that contribute to explaining the variability of QoL were not considered in the present model and should be added in future.

Table 4 shows the final regression model considering functioning and disability, measured through the WHODAS 2.0 instrument, as a dependent variable, adjusted for age and gender.

**Table 4:** Final regression model considering functioning and disability as dependent variable, measured through the WHODAS 2.0 instrument, adjusted for age and gender.

**Variable**	**Coefficient**	**Standard error**
Mobility score (0 = bad – 100 = best)	0.547***	0.020
Loneliness score (0 = best – 100 = bad)	0.046***	0.013
Social Support score (0 = bad – 100 = best)	−0.026*	0.014
Constant	7.414**	2.935
No. of Observation	380	
*R*^2^ (Adjusted *R*^2^)	0.69 (0.69)	

*WHODAS, World Health Organization Disability Assessment Schedule.*

*** p <0.01, ** p <0.05, * p <0.1.

In this model, the most significant predictors of disability were found to be Loneliness and Mobility Scales. In particular, an increase in loneliness significantly predicted a higher disability level and a reduction in mobility predicted an increase in disability. Social support also emerged as a significant predictor of disability, with higher levels of social support predicting lower levels of disability.

For the present model, two subjects were considered outliers, as their scores were too high compared to the rest of the sample and were therefore discarded from the analysis.

## Discussion

The present study investigated the role of social networks and built environment as predictive factors of quality of life and disability in a sample of older adults in Italy. The results showed that higher scores in the social network index and social support predicted an increase in the quality of life and the reduction of disability, respectively. On the contrary, loneliness significantly predicted a reduction in the quality of life and an increase in disability levels. Moreover, our results showed that a reduction in the mobility levels predicted an increase in disability, and an increase in disability further predicted a decrease in quality of life.

Our results showed that social network significantly predicts quality of life. These results are in line with previous evidence that highlighted the positive impact of social networks on the quality of life (42, 43). This was reported also in the COURAGE in Europe study, where the results showed that the presence of wide and rich social networks, good social participation, and strong social support have a positive effect on QoL (27). Other studies also documented the positive impact of social networks on health, wellbeing, quality of life, and life satisfaction (24, 25, 28, 43).

Our study showed the importance of both the quantity and the quality of social relationships. This evidence reinforces the findings of different studies that highlighted the importance of not only the size of social networks but also the quality of the relationships (social support) for wellbeing and life satisfaction (9, 25, 43). Indeed, social networks can include various types of relationships, such as family members, close friends, neighbors, and acquaintances. In fact, the number of social ties tends to restrict with age. Older adults usually have fewer peripheral contacts compared to younger adults; however, meaningful and more supportive relationships tend to remain stable and are important in later life (9, 24).

Our results also showed the role of social support in reducing disability, indicating that the presence of social support can significantly contribute to maintaining higher functioning in later life. Social support and help from significant relationships can become important facilitators in late life, when physical limitations and health issues are more likely to emerge. Many studies outline how friendship relations can be very important for the wellbeing of the older adult population; in this context, relations with friends and neighbors can assume great importance to provide social support, particularly for people living alone or in the absence of other social bonds (24, 25, 29).

Finally, our study showed that loneliness significantly predicts the quality of life and disability. This result confirms the evidence from different studies regarding the significant effect of loneliness on health and QoL (9, 26, 29). The study of Liu and colleagues found a positive relationship between loneliness and depression among elderly people, thus confirming a decrease in perceived QoL (28). Another study conducted on the elderly in Sweden and Spain showed that social isolation was negatively associated with QoL. However, specifically in Spain, the association was stronger in those under the age of 65 years and in those with no reported chronic diseases (26). On the other hand, a disability could affect social participation, resulting in increasing loneliness. For example, a study investigating the relationship between disability and loneliness found that disability has a significant indirect effect on loneliness, and its role is mediated by social resources (44).

In the present study, the built environment has been reported as a facilitator by the majority of participants and was not found to be a significant predictor of either quality of life or disability. This result is not in line with the existing evidence that reveals a significant role of the built environment in promoting functioning (7, 11, 16, 22, 45). Although our sample was quite homogeneous, mostly having a property house, living in cities, and not having many architectural barriers either in the homes or in the external environment, a possible explanation for this finding can be the difficulty for everybody, including older adults, to identify barriers in their own environment, especially in their houses. Some studies show how the elderly do not often perceive obstacles in their living environment. Moreover, the evaluation of the external environment could be influenced by the restrictions imposed during the COVID-19 period that significantly limited movements and community participation.

However, our results showed a significant correlation between mobility and functioning, with a reduction in mobility predicting an increase in disability. It is well-known that a reduction in mobility influences health and functioning, thus contributing to increased disability (15, 46–48). The characteristics of the built environment are essential to perform some activities; therefore, an accessible and facilitating built environment can support mobility and hence contribute to preventing or reducing disability. Indeed, since the elderly spend most of their time in the home and neighborhood, the presence of obstacles in these environments can constitute a barrier to mobility and contribute to a deterioration in functioning (11, 16). Conversely, some elements of the built environment, such as the presence of parks and pedestrian areas, adequate parking facilities, and efficient public transport, can favor mobility and thus reduce disability in older adults (18).

Moreover, a reduction in mobility can create difficulties in attending community activities and in reaching the places where it is possible to meet people and build social relationships, also considering that a reduction in mobility also often affects the friends of older adults, usually of the same age group (9). This aspect can be particularly relevant in large cities, because of their territorial dispersion and the consequent probability that one's relational circle does not live nearby.

Our study presents some limitations. First, the sample size is limited and composed of people who work as volunteers and belong to a senior association, and people who have the willingness to stay together and remain active. Therefore, they can be considered as people with a good level of activity and social engagement. In addition, the sample is constituted of people having a good level of education and good health status. For these reasons, caution is needed in the generalization of these preliminary results, also considering the small predictive power of the variables investigated. Indeed, we hypothesized that the existence of variables that we have not considered in our study and inserted in our model can influence the variance of the dependent variable.

Moreover, the historical period during which the study was conducted has to be considered. Indeed, a part of the data collection was performed before the advent of the COVID-19 pandemic (82 interviews), and the remaining part (349 interviews) during the pandemic and lockdowns. Therefore, other factors related to the pandemic and social restrictions need to be considered in the interpretation of the results.

In conclusion, our results strengthen the importance of social networks for the functioning and quality of life in elderly people. It needs to be considered that older adults are at higher risk of loneliness than younger adults (28). Our results, in line with previous evidence, confirm that supportive social networks can be a protective factor for older people and prevent consequences in terms of disability and impaired quality of life (28). Being involved in associations or other social groups can be helpful for older adults to build relationships and expand the size of their social network (23). This element has also been highlighted in the WHO global report on Decade of Healthy Aging (5) and is one of the pillars that politicians should consider: How can social networks be supported in terms of policies and supportive actions?

Particularly for those countries that have an increasingly aging population, it becomes really important to explore the quality of life and disability in older adults by identifying facilitators and barriers that could be the target for actions and modifications if needed, so as to put into practice the actions that enable this segment of the population to maintain the best life possible for as long as possible. Indeed, following the bio-psychosocial perspective, quality of life and disability depend both on the health condition of the individual and on the features of the environment in which people live (5). Older adults are more likely to suffer from one or more chronic health issues, a condition that concerns the intrinsic capacity of an individual and is often hardly modifiable. On the contrary, the social and physical/architectural environmental factors can be addressed and effectively modified with appropriate interventions (7, 30). Acting on the environmental factors is crucial to promote and maintain the functional ability of older adults, even in the presence of some physical limitations.

The interaction between health status and environmental factors, and their effects on subjective dimensions, such as quality of life, is useful information for policymakers to plan actions addressed to face emerging problems and aimed at supporting the aging population (30). Government and stakeholders should dedicate resources to assess and monitor the active and healthy aging process, considering the different factors that contribute to the quality of life and disability among this population (5).

Interventions aimed at improving functioning and quality of life in older adults need to consider the crucial role of the social networks. At the community level, there is a need for actions to promote the possibility for older adults to meet people and expand their social bonds. Senior associations play a crucial role in promoting social connection and mutual support among older adults, in addition to providing assistance and support for people with reduced mobility and health conditions or in the condition of marginalization. Associations should develop and encourage opportunities for social participation to favor socialization and contrast isolation in the aging population. Moreover, urban and architectural designers should take care to design neighborhood environments that facilitate relationships, such as small, well-kept green areas adjacent to homes or common areas in large city condominiums where relationships with neighbors can be built.

At both research and policy levels, resources should be devoted to the identification of strategies and concrete effective actions that can promote the creation and consolidation of social relations in the elderly population, contrasting isolation. Healthy aging is achievable if all concur to be and to create facilitating environments.

## Data Availability Statement

The raw data supporting the conclusions of this article will be made available by the authors, without undue reservation.

## Ethics Statement

The studies involving human participants were reviewed and approved by Fondazione IRCCS Istituto Neurologico Carlo Besta. The patients/participants provided their written informed consent to participate in this study.

## Author Contributions

EG, CT, ML, RR, EB, and RC contributed to the conception and design of the study. BC organized the database and performed the statistical analysis. EG and CT wrote the first draft of the manuscript. ML, RR, EB, RC, CF, and SB contributed to the revision and the approval of the submitted version. All authors contributed to the article and approved the submitted version.

## Funding

The TAPAS in Aging project (Time and Places and Space in Aging) was funded by Fondazione Cariplo (Project ID: 2018-0859).

## Conflict of Interest

The authors declare that the research was conducted in the absence of any commercial or financial relationships that could be construed as a potential conflict of interest.

## Publisher's Note

All claims expressed in this article are solely those of the authors and do not necessarily represent those of their affiliated organizations, or those of the publisher, the editors and the reviewers. Any product that may be evaluated in this article, or claim that may be made by its manufacturer, is not guaranteed or endorsed by the publisher.
